# Small lymphocytic lymphoma with florid perniosis-like features: a case report

**DOI:** 10.1186/s12895-015-0032-z

**Published:** 2015-07-23

**Authors:** Taylor M. Morris, Rosetta Mazzola, Brian Berry, Douglas Sawyer, David L. Saltman

**Affiliations:** Louisiana State University School of Medicine, 1501 Kings Hwy, Shreveport, LA 71103 USA; British Columbia Cancer Agency, 2410 Lee Avenue, Victoria, British Columbia V8R 6 V5 Canada; Department of Pathology, Island Health, Victoria, British Columbia V8R 1 J8 Canada

**Keywords:** Perniosis-like, Small lymphocytic lymphoma, Cancer, Chemotherapy

## Abstract

**Background:**

Small lymphocytic lymphoma is a relatively rare B-cell non-Hodgkin lymphoma that is considered to be the tissue equivalent of the much more common entity chronic lymphocytic leukemia. Cutaneous manifestations of small lymphocytic lymphoma are infrequent and the literature regarding them sparse. We describe here a case of a patient with a history of small lymphocytic lymphoma who developed perniosis-like features of the digits.

**Case presentation:**

An 86-year old male patient with previously diagnosed small lymphocytic lymphoma developed painful erythematous swelling of the periungual area of his fingers and toes. This was associated with a dense dermal infiltration of CD5-positive B-lymphoid cells consistent with his low-grade B-cell lymphoma. Although partially refractory to local radiotherapy, the painful swelling of the fingers and toes resolved fully following systemic therapy with chlorambucil and rituximab.

**Conclusions:**

This unusual cutaneous manifestation of a lymphoma and the favourable response to systemic therapy may be instructive for the management of other patients who develop similar perniosis-like features.

## Background

Small lymphocytic lymphoma (SLL), the tissue equivalent of chronic lymphocytic leukemia (CLL), typically presents with lymphadenopathy, organomegaly, and the presence of infiltrating monoclonal B cells having the same immunophenotype as CLL cells but lacking peripheral blood lymphocytosis [1]. The dermatological literature relating to SLL and CLL is sparse. We report a case of perniosis-like involvement of the digits in a patient with SLL and review the literature regarding cutaneous manifestations of SLL and CLL.

## Case presentation

The patient is an 86-year-old male who presented in 2004 with small nodules on both ears. There was no palpable lymphadenopathy or splenomegaly noted on physical examination. A biopsy of one of these nodules showed widespread infiltration of small, mature lymphoid cells with occasional proliferation centres. Immunohistochemistry revealed the majority of cells to be CD5, CD20 positive and CD 3 and cyclin D1 negative. The peripheral blood showed a white blood cell count (WBC) of 6.9 × 10^9^/L (4–10.5), neutrophils 4.44 × 10^9^/L (2.00-7.00), lymphocytes 1.89 × 10^9^/L (1.50-4.0), haemoglobin 145 g/L (136–170) and platelets 307 × 10^9^/L (150–400). The remainder of the leukocyte differential was unremarkable. A blood smear showed normal appearing lymphocytes with occasional reactive form but no smudge cells or plasmacytoid features. A bone marrow biopsy showed a subtle interstitial small lymphoid infiltration with mature chromatin and no plasmacytoid differentiation constituting less than 20 % of the marrow. Immunophenotyping by flow cytometry performed on the bone marrow confirmed a lambda monoclonal B-cell proliferation with dim surface immunoglobulin expression and CD5 and CD19 co-expression. CD20 expression was evident but CD10, CD23 and FMC7 staining was absent. Conventional karyotype analysis was not performed, but fluorescence in situ hybridization was negative for the CCND1-IGH fusion gene created by the t(11;14). The findings from the ear lobe biopsy, peripheral blood and bone marrow biopsy were thought to be most consistent with a diagnosis of SLL.

**Fig. 1:**
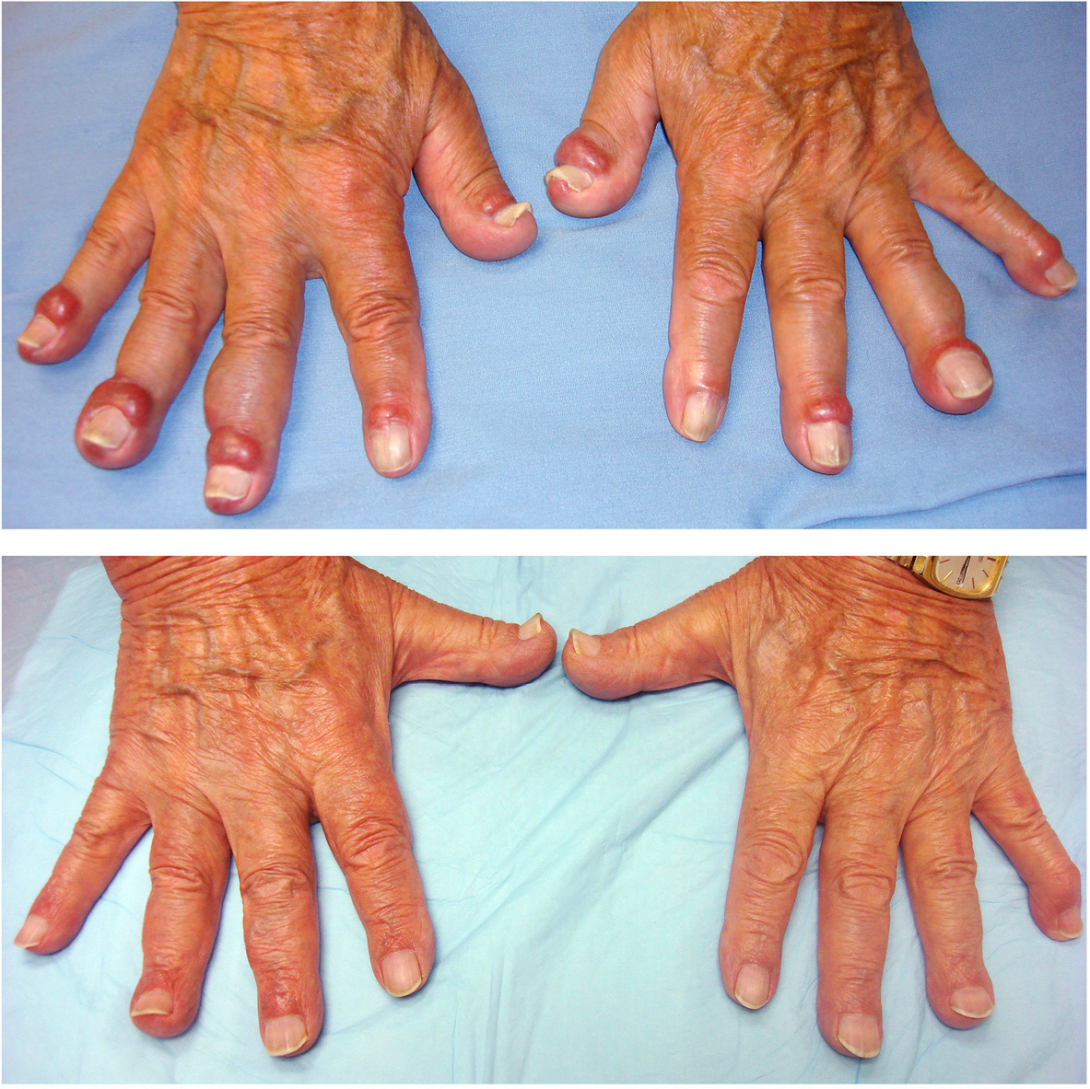
Upper panel: Bilateral periungual swelling of the fingers. Lower panel: Images of the fingers after six cycles of chlorambucil and rituximab

**Fig. 2:**
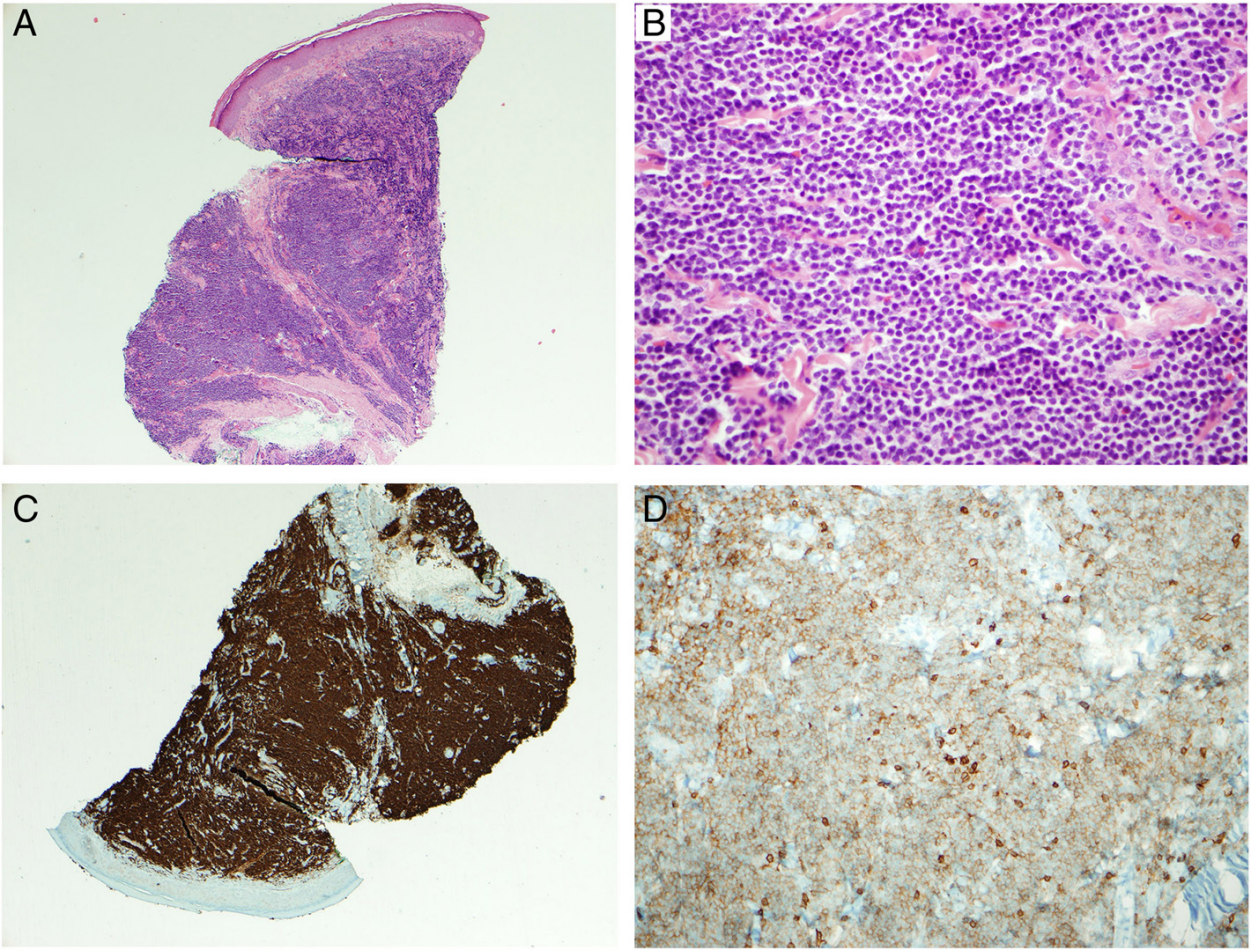
Micrograph prepared from periungual biopsy of right second finger. **a**, H&E x 40 - Low power view of micrograph prepared from periungual biopsy of right second finger, showing diffuse dermal infiltration by monomorphous lymphoid infiltrate, typical of SLL/CLL. **b**, H&E x 400 - Detail of uniform small lymphocytes with round nuclei, condensed chromatin and scanty cytoplasm. **c**, CD20 - The lesional cells are uniformly positive for CD20. **d**, CD5 - The CD20 + cells show aberrant weak positivity for CD5. Note the scattered T-cells that stain more strongly for CD5

In 2007, the patient developed jaundice and abdominal pain due to choledocholithiasis and underwent a cholecystectomy. In addition to cholelithiasis, there was a background population of B-lymphocytes co-expressing CD5 and CD20 with no evidence of CD23, CD10 or cylcin D1 expression. A liver biopsy performed the same year to investigate abnormal liver function tests showed marked lymphocytic and plasmacytic infiltration as well as reactive secondary follicles predominately involving the portal tracts, suggestive of autoimmune hepatitis but with an unusual degree of lymphocyte infiltration with the same phenotypic profile as the gallbladder. The patient was treated with azathioprine with a normalization of his liver function tests and symptoms. He was referred to the British Columbia Cancer Agency in July 2012 because of painful erythematous bulging of the periungual areas of all his fingers (Fig. 1, upper panel) and toes. Physical examination revealed palpable lymphadenopathy in the left anterior cervical chain, the axillae and the left groin, with the largest lymph gland estimated to be one centimeter in greatest diameter. Splenomegaly was not detected. A biopsy of the affected skin from the right second finger showed a dense and relatively monomorphic infiltration of the superficial and deeper dermis, by small to intermediate sized lymphocytes with condensed chromatin, irregular nuclear contours and minimal cytoplasm (Fig. 2, a and b). Immunohistochemistry confirmed the vast majority of the infiltrate to be of B-lymphoid lineage with these cells aberrantly co-expressing CD20 and CD5 (while being CD10-, 23-, 43-, and cyclin D1-negative) (Fig. 2, c and d). These features were in keeping with a low-grade B-cell lymphoma, such as small lymphocytic lymphoma. The patient received radiation to the fingers and thumbs without any significant improvement. The right third, fourth and fifth fingers were re-irradiated in September 2012 but again with only a slight improvement. A serum protein electrophoresis showed a marked polyclonal IgG and IgA hypergammaglobulinemia, with a normal IgM level of 0.74 g/L (0.5-2.00). There was a significant increase in the serum viscosity but only a mild increase in the liver aminotransferase enzymes.

The patient was started on chlorambucil and rituximab in April 2013 and received a total of 6 cycles. The serum immunoglobulin levels decreased significantly with each treatment cycle, as did the swelling and pain in the fingers (Fig. 1, lower panel) and toes. The serum viscosity level and the liver function tests returned to their normal ranges. A rise in the liver function tests in the fall of 2014 was not accompanied by any progression of the dermopathy or significant change in the serum immunoglobulins. It was treated successfully with a 3-month course of oral budesonide. The patient was last seen in February 2015, at which time there was no recurrence of the abnormalities of the digits, no progression of the gammopathy, and his serum viscosity and liver enzymes were within the normal range.

## Discussion

The patient discussed in this report developed an unusual dermatological manifestation of a low-grade B-cell lymphoma. The histology and immunophenotype of the ear, finger and bone marrow biopsies, together with the patient’s indolent course are consistent with a diagnosis of small lymphocytic lymphoma (SLL). Although CD23 expression is usually seen in CLL/SLL, CD23 negative cases are not rare [2]. The negative FMC7 and cyclin D1 expression and absent t(11;14) virtually excludes the diagnosis of mantle cell lymphoma. Furthermore, mantle cell lymphoma usually follows a more aggressive course while our patient had a very indolent course, only requiring treatment because of his painful fingers. Expression of SOX11 is seen in the majority of MCL cases [3]. If our case was clinically, morphologically and immunophenotypically suspicious for MCL but cyclin D1 expression negative, then testing for SOX11 expression may have been useful in ruling out a diagnosis of MCL. Lymphoplasmacytic lymphoma (LPL) can rarely be CD5-positive. However, our case did not show plasmacytoid cytologic features and demonstrated surface immunoglobulin expression dimmer than would be expected in LPL. FMC7 was also negative and an IgM paraprotein was never detected [2]. The appearance of the monoclonal lymphoid infiltrates seen in the biopsies without the typical clear abundant cytoplasm often observed in marginal zone lymphoma, together with the positive CD5 expression and negative FMC7 seen in this case make a diagnosis of marginal zone lymphoma less likely as well. However, CD5-postive marginal zone lymphomas have been reported [4, 5].

Dermatologic manifestations are relatively uncommon in SLL and CLL. Estimated incidences are in the range of 4 to 45 %, but most of the previously reported reviews included cases where the skin was infiltrated with B-lymphocytes and other non-lymphoid malignancies and nonmalignant disorders associated with CLL/SLL [6, 7]. The cutaneous manifestations of CLL/SLL infiltrating the skin include small or large tumour nodules, papules, plaques and erythroderma. Infiltrates may be solitary, grouped or generalized [6, 7]. With regard to the current patient, the gross appearance of his digits was similar to perniosis (chilblains). A handful of reports describing a perniosis-like presentation with leukemia cutis have appeared in the literature; however, these have typically been associated with myelomonocytic leukemias rather than lymphoid malignancies [8–11]. SLL with secondary cutaneous involvement needs to be differentiated from primary cutaneous B-cell lymphoma, which is comprised of three main types: primary cutaneous marginal zone B-cell lymphoma, primary cutaneous follicle center lymphoma, and primary cutaneous large B-cell lymphoma (leg type) [12, 13]. Primary cutaneous marginal zone B-cell and follicle center lymphoma are indolent types with excellent prognoses. These should be treated with nonaggressive therapies, whereas primary cutaneous large B-cell lymphoma (leg type) is often a rapidly growing tumour requiring aggressive chemotherapy.

In patients with SLL or CLL, Richter’s syndrome (RS), the development of high-grade NHL, can occur. RS occurs in 3-10 % of cases and may be triggered by viral infections (e.g. Epstein-Barr virus infection) or acquisition of additional genetic abnormalities [14, 15]. The disease commonly involves the lymph nodes, bone marrow and viscera, with rare skin manifestations. When cutaneous symptoms arise, they may include erythematous papules, nodules, or plaques involving the face, back, and chest. Although clinical data are limited, cutaneous RS generally has a less aggressive course with longer survival than extracutaneous RS [15].

The most commonly reported non-lymphoid malignant cutaneous conditions frequently associated with SLL/CLL consist of skin cancers (basal cell carcinoma, squamous cell carcinoma, malignant melanoma, Merkel cell carcinoma, and mycosis fungoides), viral infections (varicella-zoster, herpes simplex, and viral warts), and acute graft-versus-host disease [7, 16, 17]. Basal cell and squamous cell carcinomas as well as melanoma can develop prior to any therapeutic immunosuppression, indicating a predisposition inherent to B-cell malignancies. Perhaps this is due to the coincident abnormal T-cell function and impaired responses of B-lymphocytes to mitogens and growth factors.

Prior to diagnosis of hematologic malignancies such as SLL and CLL, exaggerated cutaneous hypersensitivity, including reactions to mosquito and other arthropod bites can be observed [18, 19]. These hypersensitivity reactions must be distinguished from lymphocytoma cutis (LC), or cutaneous lymphoid hyperplasia, the stereotypical example of the cutaneous B-cell pseudolymphoma. This can similarly be induced by various antigenic stimuli, including arthropod bites, ingestion of drugs, allergy hyposensitization injections or vaccination, chronic infections, and tattoo pigment [20, 21]. By contrast, cutaneous disease observed at sites of previous herpes simplex and herpes zoster infections in CLL/SLL patients represents specific infiltration by leukemia cells, rather than LC [22]. In endemic regions, *Borrelia burgdorferi*, the spirochete responsible for Lyme disease, is the predominant causative agent for LC and is associated most commonly with sites of involvement including the nipple, genital area, and earlobe [23]. Of note, cases of primary cutaneous B-cell lymphoma associated with *B. burgdorferi* infection have been described, and in patients with CLL this infection can trigger the development of specific cutaneous malignant infiltrates at typical sites of involvement for LC [24, 25].

An additional skin disease associated with CLL and malignant B-cell lymphomas is paraneoplastic pemphigus (PNP), a recently characterized rare autoimmune vesiculobullous eruption producing painful mucocutaneous ulcerations in patients [26]. This poor-prognosis blistering disorder is associated with antibodies targeted against proteins of keratinocyte adhesion, thereby causing acantholysis. Clinical findings include oral erosions and flaccid cutaneous bullae and erosions. Retrospective uncontrolled studies suggest that immunosuppressive agents reduce mortality in pemphigus, and the anti-CD20 monoclonal antibody rituximab may be an effective treatment for refractory patients [26]. Treating the underlying malignancy with chemotherapy may help to control autoantibody production. Interestingly, however, fludarabine, a purine analog chemotherapy agent with substantial activity in CLL and indolent NHL, may be associated with the induction of PNP in some cases [27].

Additional conditions associated with periungual swelling include acute and chronic paronychia, clubbing and pseudoclubbing, amyloid arthopathy, and the cutaneous involvement associated with lupus, scleroderma, dermatomyositis and other connective tissue diseases; however, neither the history nor clinical presentation of the patient reported herein are consistent with any of these disorders [28–31].

## Conclusions

We present a case of SLL with a previously unreported complication demonstrating perniosis-like features involving the fingers and toes. The infiltration of the periungual areas with lymphoma cells was confirmed by histopathology findings. The contribution of the polyclonal hypergammaglobulinemia and hyperviscosity to the periungual changes is not clear. The complete resolution of the perniosis-like features after treatment with chemotherapy and rituximab immunotherapy may be instructive for the management of other patients who develop this disease manifestation.

## Consent

Written informed consent was obtained from the patient for publication of this case report and any accompanying images. A copy of the written consent is available for review from the Editor of this journal.

## Abbreviations

SLL: Small lymphocytic lymphoma
CLL: Chronic lymphocytic leukemia
NHL: Non-Hodgkin lymphoma
LC: Lymphocytoma cutis
LPL: Lymphoplasmacytic lymphoma
PNP: Paraneoplastic pemphigus
RS: Richter’s syndrome
